# Emerging Roles of SKP2 in Cancer Drug Resistance

**DOI:** 10.3390/cells10051147

**Published:** 2021-05-10

**Authors:** Ting Wu, Xinsheng Gu, Hongmei Cui

**Affiliations:** 1 Institute of Toxicology, School of Public Health, Lanzhou University, Lanzhou 730000, China; wut19@lzu.edu.cn; 2 Department of Pharmacology, College of Basic Medical Sciences, Hubei University of Medicine, Shiyan 442000, China; gu.xinsheng@gmail.com

**Keywords:** Skp2, drug resistance, Akt, p27, cell cycle, mitosis, EMT, DNA damage response and repair, inhibitors

## Abstract

More than half of all cancer patients receive chemotherapy, however, some of them easily acquire drug resistance. Resistance to chemotherapy has become a massive obstacle to achieve high rates of pathological complete response during cancer therapy. S-phase kinase-associated protein 2 (Skp2), as an E3 ligase, was found to be highly correlated with drug resistance and poor prognosis. In this review, we summarize the mechanisms that Skp2 confers to drug resistance, including the Akt-Skp2 feedback loop, Skp2-p27 pathway, cell cycle and mitosis regulation, EMT (epithelial-mesenchymal transition) property, enhanced DNA damage response and repair, etc. We also addressed novel molecules that either inhibit Skp2 expression or target Skp2-centered interactions, which might have vast potential for application in clinics and benefit cancer patients in the future.

## 1. Introduction

More than half of all cancer patients will receive chemotherapy. The rate of complete pathological responses to chemotherapy remains low, partially due to acquired chemoresistance. Resistance to chemotherapy occurs in 50–96% of cancer patients who begin to obtain tumor recurrence within 6–9 months of treatment [1,2], which has become a massive obstacle in achieving high rates of complete pathological responses during cancer therapy.

Cancer cells have been found to employ multiple mechanisms to resist chemical drug-induced damage. Such mechanisms include altered cell cycles, evading apoptosis, enhanced DNA damage response and repair, remodeling of the cellular energetic system, and acquisition of resistance capability [1]. 

Thus far, numerous studies have reported that the ubiquitin–proteasome system (UPS) E3 ligase contributes to drug resistance by disturbing cell proliferation and apoptosis, cell cycle, transcription, cell mitosis, and spindle assembly [3,4,5]. UPS is the main pathway of protein degradation in cells and participates in the degradation of more than 80% of proteins in cells [6]. The degradation of proteins by UPS is a multi-step enzymatic process that includes ubiquitin-activating enzyme (E1), ubiquitin-conjugating enzyme (E2), and ubiquitin-protein ligase (E3) [6], among which, E3 ubiquitin ligase is the key enzyme that specifically and strictly recognizes substrates for ubiquitination and subsequent degradation. Based on the protein sequence, E3 ligase has been classified to three major types: HECT (homologous to the E6-AP carboxyl terminus) type, the RING (interesting new gene) finger type, and the RBR (ring between ring fingers) domain-containing ubiquitin ligases [7,8,9]. F-box proteins belong to ring finger type E3 ligases and are critically involved in tumorigenesis through governing the ubiquitination and subsequent degradation of cell cycle proteins, and finally leads to aberrant cell cycle progression and tumorigenesis, which has been reviewed in detail [7,8]. 

S-phase kinase-associated protein 2 (Skp2), also known as p45, was first cloned from human fibroblasts by Zhang et al., in 1995 [10,11]. Skp2 protein is a key component of SKP1-cullin 1-F-box (SCF) complex, which is one of the major categories of E3 ligases where the F-box protein is responsible for substrate recognition [12,13]. Structurally, Skp2 protein consists of a putative nucleus localization sequence (NLS) on the N-terminal (1–111aa), followed by a short 40aa F-box (112–152aa), linker, and leucine-rich repeat (LRR) which is essential for Skp2 to recognize its substrates [13,14,15]. Skp2 largely functions as an oncoprotein, which has been substantially reviewed in [13,15,16,17,18]. Recently, it has been reported to be associated with drug resistance in various human cancers, which suggests that Skp2 could be a better cancer therapeutic target and may overcome drug resistance. In this review, we will summarize the role of Skp2 in the development of chemoresistance and Skp2 inhibitors that show great potential to overcome resistance.

## 2. Expression of Skp2 Associated with Tumor Malignancy and Drug Resistance

Skp2 is involved in cell proliferation, apoptosis, migration, invasion, angiogenesis, and metastasis of many malignant human tumors [16,17], and is over-expressed in many types of human cancers, including breast cancer, non-small cell lung cancer [19,20], prostate cancer [21,22], pancreatic cancer [23], multiple myeloma [24], gastric cancer [25,26], melanoma [27,28,29], lymphoma [30,31,32], nasopharyngeal carcinoma [33,34], and osteosarcoma [35,36]. Over-expression of Skp2 is often associated with higher grades and a greater malignancy of tumors as well as a poor prognosis of cancers, such as breast cancer [37,38], nasopharyngeal carcinoma [33], rectal cancer treated with chemoradiotherapy [39], oral squamous cell carcinomas [40], and non-small-cell lung cancer [41], which suggests that Skp2 engages in tumorigenic activity and is a promising target for cancer therapy. 

Skp2 not only functions as a pro-survival gene in tumor progress, but also is involved in cancer-associated drug resistance. Numerous studies have reported that Skp2 contributes to paclitaxel resistance [3,42,43,44,45], cisplatin resistance [32,46,47,48], doxorubicin resistance [49], tamoxifen resistance [50,51], bortezomib resistance [52], PI3K inhibitor resistance [53,54,55,56,57], tyrosine kinase inhibitors resistance [58,59,60,61,62,63], methotrexate-resistant [64], radiation resistance [65,66,67,68], twist and castration resistance [69], mTOR inhibitors resistance [70], etc.

## 3. The Molecular Mechanism of Skp2 Involved in Cancer Drug Resistance

### 3.1. Signaling Feedback Loop between Akt-Skp2

Akt (protein kinase B) is a positive regulator of Skp2 through transcriptional, translational, and posttranslational regulation. Specifically, Akt induces Skp2 transcription in an E2F1 (E2F transcription factor 1)-dependent manner and, meanwhile, inhibits the transcriptional repressor FOXO3 recruiting to the Skp2 promoter [71,72]. The Akt/mTORC1/eIF4E axis regulates Skp2 mRNA translation during escape from contact inhibition exit (one of the hallmarks of cancer cells) and restores Skp2 oncogenic transformation. Skp2 mRNA has been found in wildtype Akt1/2 cells but not in polysomes isolated from Akt1/2 double knockout cells, showing that Skp2 mRNA translation is dependent on Akt. Akt initiate Skp2 mRNA translation through its downstream effector mTORC1 and eIF4E (eukaryotic initiation factor 4E). eIF4E binds to 5′ UTR of Skp2 mRNA and decreases Skp2 expression upon serum stimulation of contact inhibited cells [73]. One CREB (cAMP-response element binding protein)-binding region (BR) was identified within the promoter of Skp2, and the transcriptional activity of CREB was enhanced following Ser133 phosphorylation by PI3K/Akt or MAPK/ERK. Inhibition of the PI3K/Akt/CREB/Skp2 pathway significantly increased the sensitivity of K562 cells to tyrosine kinase inhibitors (TKI) Imatinib treatment [62]. Myristoylated alanine-rich C-kinase substrate (MARCKS) is a protein kinase C (PKC) substrate that has been previously reported to play a role in cell adhesion, spread, and mitogenesis. Studies have demonstrated that phosphorylated MARCKS bound to E2F1 and formed a cofactor complex in Skp2 promoter region. MARCKS promoted cell-cycle progression by facilitating Skp2 expression, suppressing p27 and potentially counteracting drug-induced cell-cycle arrest via augmenting CDK2-cyclin E activity. Importantly, MARCKS knockdown overcome bortezomib (BTZ, anti-myeloma drugs) resistance [74]. Bortezomib also known as Velcade (^®^), which has been approved by the FDA for clinical trials in the treatment of patients with multiple malignant cancers. BTZ has been shown to induce apoptosis and inhibit cell growth of many cancer cells [75].

Skp2 phosphorylation is mainly regulated by the PI3K/Akt pathway. Akt phosphorylates Skp2 on Ser72, which is required for Skp2 stabilization by interrupting interaction between Skp2 and E3 ligase APC/Cdh1 (anaphase promoting complex/ E-Cadherin), thus protecting Skp2 against degradation by APC/Cdh1. Ser72 locates into a putative nucleus localization sequence (NLS) and its phosphorylation by Akt leads to Skp2 stabilization and cytoplasmic translocation [76]. It is also reported that Skp2 can be phosphorylated on Ser64 by p38/MAPK, protecting Skp2 from proteasomal degradation by NK3 homeobox 1 (Nkx3-1), finally leading to Oct-4 (Octamer-binding transcription factor) over-expression and contributing to tamoxifen-resistance in MCF-7 breast cancer cells [50]. Additionally, Skp2 interacts with mTOR and mTOR complex 1 (mTORC1) also phosphorylates Skp2 on Ser64 and stabilizes Skp2 by blocking ubiquitin/proteasome pathway [77]. mTORC1 also regulates Skp2 protein translation via S6K1 and 4EBP1 [70]. Overall, Skp2 levels are a key determinant of antitumor responses to mTOR inhibitors, such as Rapamycin, highlighting a potentially critical pharmacological marker to predict sensitivity to rapamycin and Skp2 silencing strategies for therapeutic purposes. There are two major phosphorylation sites on Akt, in which mTOR complex 2 (mTORC2) controls cell survival following phosphorylation of Akt on Ser473 and PDK1 (3-phosphoinositide-dependent protein kinase 1) phosphorylation on Thr308, which is necessary for the activation of Akt. Downregulation of Skp2 by rapamycin is phosphorylation dependent, in which dephosphorylation of Akt at Ser473 and consequently decreased Skp2 expression [70]. AMPK (AMP-activated protein kinase) also contributes to Skp2 phosphorylation. Skp2 S256 phosphorylation by AMPK promotes Skp2-SCF complex formation enhancing its E3 ligase activation, which leads to K63-ubiquitination and activation of Akt, and finally contributing to a resistance to EGFR targeting therapy, such as Gefitinib, in non-small cell lung cancer cells (NSCLC) [57]. The results of preclinical studies and early clinical trials showed that gefitinib (‘Iressa’, ZD1839), an EGFR-tyrosine kinase inhibitor, up-regulates p27KIP1 and induces G1 arrest in oral squamous cell carcinoma cell lines [78]. 

It is well-known that Akt is a Skp2 substrate undergoing K63-linked polyubiquitination, which leads to Akt activation [79]. Skp2 ubiquitinate both Akt1 and Akt2, but not Akt3, and Skp2 is critical for EGF-mediated Akt ubiquitination and membrane recruitment. K8 and K14 residues within the PH domain on Akt are target sites for Skp2, which are required for Akt membrane recruitment and activation. Additionally, Skp2 knockdown suppressed glucose uptake and glycolysis by reduced Glu1 transcription and protein expression in breast cancer cells upon EGF or HRG stimulation, repressing breast cancer development. Therefore, Skp2 serves as a marker for poor prognosis in Her2-positive patients. Skp2 silencing sensitizes Her2-over-expressing tumors to Herceptin treatment [79]. Skp2-mediated K63-linked ubiquitination on Akt also enhances the interaction between Akt and mitochondrial Hexokinase 2 (HK2) and eventually increases HK2 phosphorylation on Thr473 and mitochondrial localization. HK2 interacts with Akt in mitochondria, which indicates that depletion of Skp2-promoted cisplatin-induced apoptosis is related to HK2 phosphorylation and mitochondrial localization. Depletion of Skp2 sensitized cells to cisplatin treatment, highlighting a promising approach for Skp2 targeting therapy in nasopharyngeal carcinoma (NPC) treatment [46].

**Figure 1:**
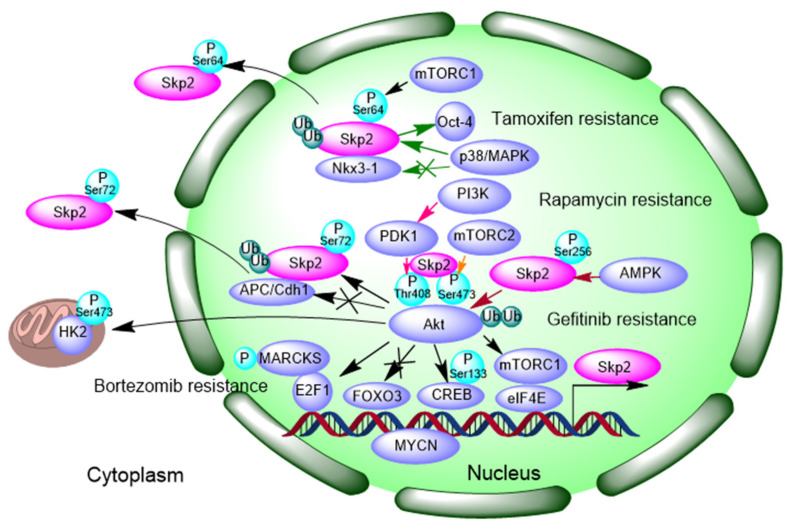
Positive feedback loop of Akt-Skp2 contributes to Tamoxifen resistance, Rapamycin resistance, Bortezomib resistance and Gefitinib resistance. Colorful arrows indicate different signaling pathways. P: phosphorylation; Ub, Ubiquitination. Skp2: S-phase kinase-associated protein 2, NKX3-1: NK3 homeobox 1, OCT-4: Octamer-binding transcription factor, mTORC1: mTOR complex 1, mTORC 2: mTOR complex 2, PI3K: phosphatidylinositol-3-kinase, PDK1: 3-phosphoinositide-dependent protein kinase 1, Akt: protein kinase B, APC/CDH1: anaphase promoting complex/ E-Cadherin, MARCKS: Myristoylated alanine-rich C-kinase substrate, FOXO3: Forkhead box O3, E2F1: E2F transcription factor 1, CREB: cAMP-response element binding protein, EIF4E: eukaryotic initiation factor 4E, HK2: Hexokinase 2, AMPK: AMP-activated protein kinase, MYCN: N-myc proto-oncogene protein.

These findings indicate that Akt and Skp2 correspond to each other enhance its function activation through a positive feedback loop (Figure 1). Emilie et al., found that inhibition of PI3K (phosphatidylinositol-3-kinas) promotes Akt reactivations as evidenced by elevated p-Akt (Ser473, Thr408) expression, which is dependent on accumulation of Skp2. PI3K resistance correlates with the Skp2-Akt signaling feedback mechanism [55].

### 3.2. Negative Modulating Substrates p27 and Contribution to Autophagy

CKIs (CDK inhibitors), p21 and p27, promote cell cycle arrest and/or delay the anti-mitogenic response by inhibiting CDK activity. Mitogenic growth factor signaling causes loss of p27, and p27 levels and/or activity increase in response to differentiation signals. p27 is a cyclin-dependent kinase inhibitor, which mainly drives cells from the G1 to the S-phase of the cell division cycle. CDK2-dependent phosphorylation of p27 on threonine 187 (Thr187) is required for its ubiquitination and for its degradation [80]. p27 Thr187 mutant cannot be bound by Skp2 owing to the loss of Thr187 phosphorylation [81,82]. p27 mRNA levels are constant throughout the cell cycle and p27 protein levels are regulated by E3 ligase Skp2-mediated proteolysis. That p27 is ubiquitinated in a phosphorylation dependent manner suggests an SCF-dependent ubiquitination [80,83]. 

Coupling opposite trend between Skp2 and p27 levels has been found in several cancer types, including hepatocellular carcinoma [84], gastric carcinoma [85], and lung cancer [44]. PPARγ ligand troglitazone treatment induced accumulation of p27 and cyclin E, paralleled with decreased Skp2 expression. Over-expressed Skp2 confers troglitazone resistance in hepatoma cells [84]. Phosphorylation of p27 at Thr187 and Ser10 is a prerequisite for its degradation via the E3 ubiquitin ligases Skp2 (nucleus) and KPC (Kip1 ubiquitylation-promoting complex) (cytoplasmic), respectively. Additionally, Ser10-phosphorylated p27 is predominantly localized within the cytoplasm, mediated by nucleus export protein CRM1 (chromosome region maintenance 1). Another E3 ubiquitin ligase, PIRH2 (p53-induced RING-H2), degrades p27 in both the cytoplasm and nucleus independent of phosphorylation state. Phosphorylation of p27 at Thr187 promotes Skp2-dependent degradation in the nucleus. As such, inhibition of cell cycle entry and progression to induce apoptosis in a variety of cancers may be achieved with strategies designed to correct p27 localization and/or block its degradation [12]. The CKI protein p27 negatively regulates the transition from the G1 to the S phases of the cell cycle by inhibiting CDK2-cyclin E and CDK2-cyclin A complexes. Hao et al., have proved that Thr187 phosphorylation can trigger the proteolysis of p27 by CDK2-cyclin E/A kinase complexes, resulting in activation of CDK-cyclin and in turn promoting G1/S phase transition [86]. Further study indicated that the phosphorylated Thr187 side chain is recognized by the Cks1 (cyclin kinase subunit 1) phosphate binding site, and Cks1 is required for p27 ubiquitination, which is essential for the G1/S transition during the cell cycle [86]. Over-expression of Cks1 increases the radiotherapy resistance in esophageal squamous cell carcinoma [67]. 

Correspondingly, transfection with Skp2 siRNA could reduce Skp2 protein and induce the accumulation of p27 protein in MCF-7 cells, inhibited the MCF-7 cell proliferation in vitro and tumor growth in vivo [87]. Targeting specific E3 ligase of p27, Skp2, results in p27- and Skp2-mediated cell-cycle arrest and activation of autophagy [88]. Tenascin-C is K63-ubiquitinated by Skp2, particularly at K942 and K1882, thus promoting its recognition by p62 and leading to its selective autophagic degradation [89]. Knockdown of Skp2 lead to the upregulation of p21 and p27 and increased sensitivity of ovarian cancer cells to Quinacrine (QC) treatment. Transient transfect with p62 plasmid rescued the QC-induced Skp2 downregulation suggesting the positive regulation of Skp2 by p62 [90].

**Figure 2:**
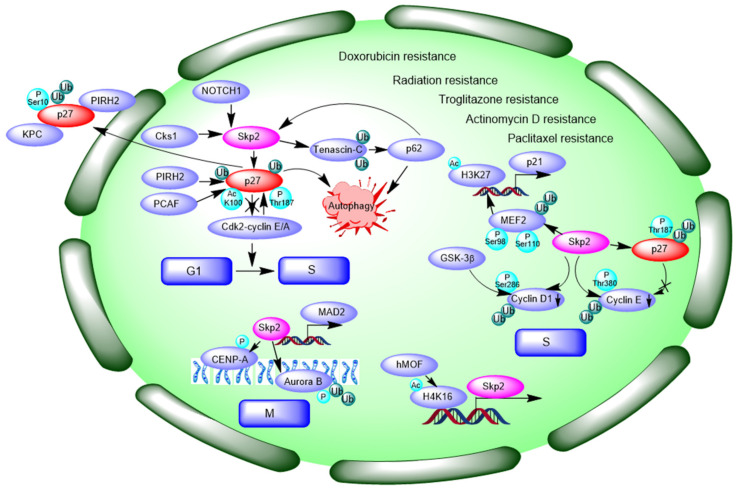
The negative regulation of p27 by Skp2 involves into cell cycle transition, and contributes to DOX resistance, Radiation resistance, Troglitazone resistance, Actinomycin D resistance and Paclitaxel resistance. Black arrows indicate different signaling pathways. P: phosphorylation; Ac: Acetlation; Ub, Ubiquitiantion. PIRH2: p53-induced RING-H2, KPC: Kip1 ubiquitylation-promoting complex, Cks1: cyclin kinase subunit 1, PCAF: p300/CBP-associated factor, MEF2: Myocyte enhancer factor 2, GSK-3β: glycogen synthase kinase 3 beta, hMOF: KAT8 or MYST1, CENP-A: centromere protein-A, MAD2: mitotic arrest deficient 2, NOTCH1: Notch homolog 1.

It has been found that Skp2 deficiency leads to the apoptosis of LSK bone marrow cells following treatment with chemotherapy agents, such as cyclophosphamide (CPA), 5-fluorouracil (5-FU) and doxorubicin (DOX). In line with this, Skp2 knockdown enhanced apoptosis in K562 and KBM5 leukemia cells with BCR-ABL oncogene over-expression upon treatment of CPA and 5-FU [91]. Over-expression of Skp2 is related to resistance to preoperative DOX-based chemotherapy in primary breast cancer [49]. Detailed analysis has shown that Skp2 knockdown leads to a significant increase in tumor necrosis factor-related apoptosis-inducing ligand TRAIL-induced apoptosis fraction and caspase activation [92]. Harada et al., have already reported that Skp2 antisense treatment induced apoptosis cascade events, including an increase in early apoptosis, fragmentation of nuclei, and activation of caspase 3/8/9 in oral cancer cells [93]. Recently, it was reported that NOTCH1/FABP7 (Notch homolog 1) targets could be Skp2 and its substrate p27. Inhibition of NOTCH1 markedly decreased Skp2 levels in in CD133+ and CD133− cells. NOTCH inhibitors show promise in neuroblastoma and brain tumors and may be used as therapy for adenoid cystic carcinoma (ACC) [65]. p27 directly binds to the transcriptional coactivator p300/CBP-associated factor (PCAF) in a region spanning amino acids 91–120. PCAF associates with p27 through its catalytic domain and acetylates p27 at K100. Acetylation of K100 promotes the stability of p27 [94]. Depletion of Skp2-induced apoptosis of drug resistant cancer cells and could be a promising method to overcome drug resistance. One study claimed that Skp2 over-expression is associated with resistance to DOX-based chemotherapy, but not to docetaxel; however, other studies demonstrated that Skp2 confers resistance to paclitaxel [49]. p27 expression was inversely correlated with Skp2 expression in paclitaxel resistant DU145-TxR or PC-3-TxR cells. Moreover, p27 was found to be upregulated in both Skp2 silencing PC-3-TxR and DU145-TxR cells. These results suggest that Skp2-p27 is associated with prostate cancer cell resistance to paclitaxel. Skp2 may be a potential therapeutic target for drug-resistant prostate cancer [43]. We summarize the Skp2-p27 regulation pathway in Figure 2.

### 3.3. Interaction and Ubiquitination of Cyclin Proteins and Control of Cell Cycle

As mentioned above, ubiquitin dependent degradation of the CDK inhibitor p27 triggers activation of CDK2-cyclin E and the G1/S cell-cycle transition, in which phosphorylation of threonine 187 (Thr187) in p27 facilitate its interaction and degradation by Skp2. Further study demonstrated that during S phase, the levels of cyclin E decreases because Skp2 binds selectively and specifically to the phosphorylated Thr380 in cyclin E and promotes cyclin E ubiquitination and degradation. Although both p27 and cyclin E can be substrates of E3 ligase Skp2, their degradation can be sequentially separated in the cell cycle. The phosphorylation of p27 on the critical Thr187 has been shown to occur in the late G1 phase, whereas cyclin E phosphorylation on Thr380 is auto-phosphorylation. Furthermore, ubiquitination of p27 requires binding to CDK2-cyclin E. p27 may inhibit the phosphorylation of cyclin E on Thr380. Therefore, cyclin E may resist Skp2 degradation. Further investigation is required to determine the exact mechanism by which Skp2 modulates the stability of various substrates to promote both the G1/S transition and S-phase progression [95].

In addition, Skp2 is implicated in cyclin D1 ubiquitination. Cyclin D1 is strongly implicated in mammary oncogenesis. Cyclin D1 accumulation is normally tightly regulated, but over-expression of cyclin D1 occurs in almost 50% of human breast cancers. Phosphorylation of cyclin D1 on Thr286 by glycogen synthase kinase 3β (GSK-3β) facilitates cyclin D1 for ubiquitination. Skp2 is implicated in the ubiquitination of cyclin D1 and cyclin D1 levels are negatively correlated with Skp2 since expression of Skp2 antisense induces accumulation of cyclin D1, and defective cyclin D1 degradation within the SK-UT-1B uterine tumor cell line are often rescued by stable transfection of Skp2. Furthermore, TSA-induced Skp2 up-regulation results in increased cyclin D1 polyubiquitination, nucleus export, and degradation within both the nucleus and the cytoplasm of MCF-7 cells [96]. 

Skp2 also plays a critical role in cell mitosis and spindle assembly checkpoint (SAC). Skp2 is essential for activation of aurora kinase B, a protein kinase that attaches the mitotic spindle to the centromere and is critical to mitosis. Phophorylation of Aurora B increases expression of phospho-CENP-A (centromere protein-A). Skp2 directly interacts with Aurora B and triggers Aurora B K63-linked ubiquitination. Skp2 knockdown cells spend more time progressing into the anaphase compared with controls, resulting in more cells remaining on mitosis, and this effect is dependent on Aurora B [97]. Skp2 positively regulates mitotic arrest deficient 2 (MAD2) and increases the phosphorylation of Rb at Ser780 and Ser807/811. Skp2 promotes the gene transcription of MAD2 via p27-CDKs-E2F1 signaling. Inhibition of Skp2 by SMIP004 sensitizes lung cancer cells to paclitaxel in A549 and NCI-H1975 cells [44]. 

Histone acetyltransferase hMOF (also called KAT8 or MYST1) promotes the S phase by regulating Skp2 mRNA expression through promotion of H4K16 acetylation. hMOF binds to the 3′UTR region of the Skp2 gene during cell cycle progression and induces H4K16 acetylation, which increases mRNA and protein expression of Skp2, thereby causing more cells to be distributed in the S phase. TSA also increases H4K16 acetylation and Skp2 mRNA expression [98]. Skp2 also interacts and mediates the ubiquitylation of MEF2C/D (myocyte enhancer factor 2 C/D), decreasing its half-life. MEF2C and MEF2D have a binding region within the first intron of p21/CDKN1A, and increased H3K27 acetylation presents epigenetic markers of open chromatin. Following the initial engagement in the G0/G1 transition, MEF2C and MEF2D must be ubiquitylated and degraded within the G1 progression to sequester the transcription of the CDKN1A gene, thus favoring entry into the S phase. Phosphorylated MEF2D on serine residues 98 and 110 enhances binding with Skp2 and facilitates its subsequent degradation and ubiquitination, thus favoring entry into the S phase [99]. We summarize how Skp2 controls cell cycle progression in Figure 2.

### 3.4. Promotion of DNA Damage Response and Repair

**Figure 3:**
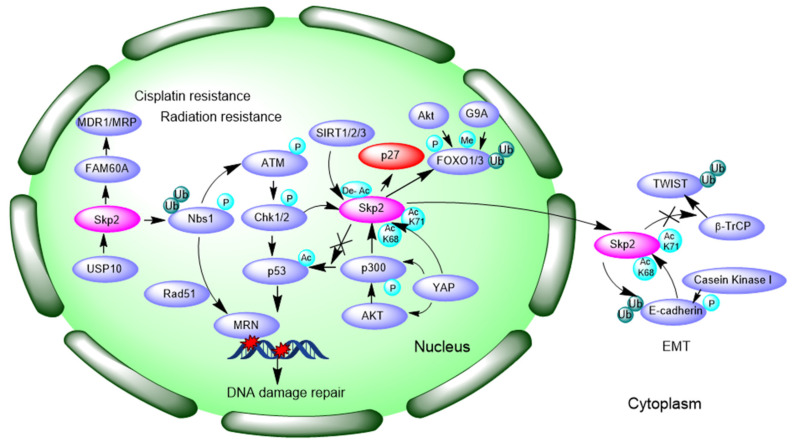
Over-expression of Skp2 contributes to cisplatin resistance and radiation resistance by enhanced MDR expression, DNA damage repair and EMT property. Black arrows indicate signaling pathways. P: phosphorylation; Ac: Acetylation; De-Ac: Deacetylation; Me: Methylation; Ub, Ubiquitination. MDR1: multi-drug resistance protein 1, MRP1: multi-drug resistance associated protein, FAM60A: family with sequence similarity 60A, USP10: ubiquitin specific peptidase 10, SIRT: sirtuin, MRN: Mre11-Rad50-Nbs1, CHK1: checkpoint kinase 1, G9A: euchromatic histone lysine methyltransferase 2, FOXO: forkhead box O, YAP: yes-associated protein, ATM: ataxia-telangiectasia mutated gene, TWIST: twist family bHLH transcription factor, β-TrCP: beta-transducin repeats-containing proteins, EMT: Epithelial-Mesenchymal Transition.

As Figure 3 shows, Skp2 can interact with key regulators in DNA damage response and repair, which may influence drug sensitivity. Radiation-induced Skp2 expression and a negative correlation with survival rates of patients were found in esophageal squamous cell carcinoma patients. Additionally, induced expression of Skp2 and paralleled Rad51 expression promoted radiation resistance of esophageal squamous cell carcinoma EC9706 cells [66].

Wu et al., have already proposed that Skp2 interacts with NBS1 and triggers K63-linked ubiquitination of NBS1 during DNA double-strand breaks, thereby promoting Ataxia-telangiectasia-mutated (ATM) recruitment to the DNA foci for its activation by MRN (Mre11-Rad50-Nbs1) complex [100]. Consistently, another study reported that the depletion of Skp2 decreases phosphorylated ATM protein levels in cisplatin-resistant mantle cell lymphoma JeKo-1 cell line and the downstream-acting factors of ATM that directly participates in DNA repair. Evidently, Skp2-deficiency is more effective in increasing cisplatin cytotoxicity in cisplatin-resistant cells than the inhibition of ATM, suggesting Skp2 is likely to be a more promising target than ATM in the treatment of cisplatin-resistant mantle cell lymphoma (MCL) [32]. Prexasertib, an inhibitor of CHK1/2 (checkpoint kinase 1/2), reduced NOTCH signaling and Skp2, enhanced the in vitro and in vivo response of head and neck squamous cell carcinoma to cisplatin and radiation, in line with another study that claimed that Skp2 is a downstream target of NOTCH signaling [48,65]. 

p300 is a histone acetylate transferase. Skp2 forms a complex with p300, and is acetylated at K68 and K71 within its NLS region, leading to increased Skp2 stability and oncogenic function, and promotes its translocation from nucleus to cytoplasm. Akt can phosphorylate and activate the acetyltransferase p300 to enhance Skp2 acetylation, cytoplasmic translocation, and protein stability [101]. p53 acetylated by p300 was viewed as an apoptotic marker; however, Skp2 suppresses p53-dependent apoptosis by interrupting interaction between p53 and p300, thereby perturbing p300-mediated p53 acetylation and apoptosis, which makes Skp2 a potential molecular target for cancer therapy [102]. Hippo pathway effector Yap (yes-associated protein) promotes diploid–polyploid conversion and polyploid cell growth through the Akt-Skp2 pathway. Yap significantly induces p300-mediated acetylation of Skp2 via Akt signaling, leading to mitotic arrest and subsequent cell polyploidy. Additionally, the pro-apoptotic factors FOXO1/3 are overly degraded by acetylated Skp2, resulting in polyploid cell division, genomic instability, and oncogenesis. Cytoplasmic Skp2 potentiates polyploidy cell proliferation and division [103]. SIRT2/3 may induce Skp2 deacetylation and subsequent degradation to abolish the effects of Skp2 on p27 and increase the expression of tumor suppressor p27 to affect NSCLC cell growth [104]. 

### 3.5. Inducing Epithelial–Mesenchymal Transition (EMT)

Epithelial–mesenchymal transition (EMT) is a biological process in which polarized phenotype epithelial cells transform into mesenchymal cells through loss of epithelial cell–cell junctions and actin cytoskeleton reorganization [105]. EMT is involved in acquired resistance to chemotherapeutic drugs in human malignancies [106]. Once the EMT property is gained, cells lose the expression of epithelial cell markers, such as E-cadherin, but gain the expression of stromal cell markers, such as N-cadherin, Slug, Snail, Twist (twist family bHLH transcription factor), Vimentin, etc. [107]. It was reported that TGF-β1 treatment increased Skp2 expression and was accompanied with increased phosphorylation of Akt1 and c-Myc protein accumulation during EMT. c-Myc specifically binds to the promoter of Skp2 and facilitates Skp2 transcription [108]. The translocation of Skp2 from nucleus to the cytoplasm enhances cellular migration through ubiquitination and destruction of E-cadherin. E-cadherin phosphorylation by CKI promotes its interaction with Skp2 [101,109]. Acquisition of EMT is associated with Skp2 expression in paclitaxel-resistant (PR) breast cancer cells, and Skp2 modulates molecular markers of EMT, including E-cadherin, Vimentin, Snail, and Slug, to enhance tumor metastasis. Skp2 siRNA partially reverses the EMT phenotype by inhibiting cell attachment and detachment capacity in PR cells [42]. Methotrexate (MTX) is an anti-folate drug used as a pivotal chemotherapeutic agent in the treatment of osteosarcoma. Stable knockdown of Skp2 abrogates EMT-induced methotrexate (MTX) resistance in osteosarcoma cells [64]. 

Importantly, Twist is over-expressed in castration-resistant tumor cells and is known to be a key driver of EMT and acquisitions of cancer stem cells. An in vivo ubiquitination study showed that Skp2 stabilized Twist protein expression by preventing proteasomal degradation of Twist by β-TrCP (beta-transducin repeats-containing proteins). Further experiments revealed that Twist over-expression augments CSC self-renewal and population, whereas Skp2 inhibition reverts Twist’s effects on CSC regulation. Furthermore, genetically depleting, or pharmacologically inactivating, Skp2 synergistically re-sensitized CRPC cells toward chemotherapies like paclitaxel or DOX. When Skp2 was knocked down in PC3 cells, E-cadherin mRNA levels were increased, whereas stromal cell markers N-cadherin, Vimentin, and S100A4, were significantly reduced. In line with a previous study, Skp2 knockdown augmented 22Rv1 CRPC cell sensitivity to paclitaxel treatment, and compound #25 (C25) substantially heightened cytotoxicity of CRPC cells [69,110]. We summarized Skp2-mediated EMT characterization in Figure 3.

### 3.6. MYC Regulates Skp2 mRNA Transcription

As mentioned above, c-Myc transcriptionally increases Skp2 mRNA levels whereas Myc protein was ubiquitinated by Skp2 via the MB2 domain [108,111]. Skp2 is also highly expressed in melanoma tissues and BRAFV600E inhibitor vemurafenib suppressed Skp2 expression and cell growth. Mechanistic studies have shown that Skp2 inhibition of BRAFV600E is dependent on c-Myc transcription factors by specifically binding to the E-box region on the Skp2 promoter, suggesting that targeting Skp2 might be a promising treatment in BRAF inhibitor resistant melanomas [112]. Evans et al., also found that MYCN (N-myc proto-oncogene protein) directly binds to the E-box in the Skp2 promoter and induces transcriptional activity, suggesting that Skp2 is a direct MYCN target gene. However, MYCN protein expression is not correlated with Skp2 protein expression [113].

### 3.7. FOXO1 and FOXO3 Interact with Skp2

Forkhead transcription factors FOXO1 (Forkhead box O1) and FOXO3a (Forkhead box O3a) play a critical role in tumor suppression by inducing growth arrest and apoptosis. Skp2 antagonizes the tumor suppression function of FOXO1 by promoting ubiquitination and degradation of the FOXO1 protein. Akt-specific phosphorylation of FOXO1 on Ser256 is prerequisite for interaction and degradation of FOXO1 by Skp2 [114,115]. FOXO1 methylation by G9a (euchromatic histone lysine methyltransferase) promoted poly-ubiquitination of FOXO1 and its proteasomal degradation via increased interaction with Skp2 [116]. Discoidin domain receptor 2 (DDR2), a mesenchymal cell-specific collagen receptor tyrosine kinase increased phosphorylation of FOXO1 in an ERK1/2 MAPK-activated manner, and further transcriptionally upregulated Skp2 and successively facilitated post-translational degradation of p27, which is essential for G1/S transition in cardiac fibroblasts [63]. 

FOXO3a is a transcriptional repressor of Skp2 that directly binds to the Skp2 promoter, thereby inhibiting Skp2 transcription. FOXO3a also directly interacts with Skp2 and disrupts Skp2-SCF complex formation, successively inhibiting Skp2-SCF E3 ligase activity and promoting p27 stability, which inhibits Skp2-mediated cell proliferation [72]. E3 ubiquitin ligase subunit Skp2 binds preferentially to deacetylated FOXO3, in which SIRT1 or SIRT2-induced FOXO3 deacetylation facilitates its ubiquitination and subsequent proteasomal degradation. Over-expression of Skp2 causes poly-ubiquitination of FOXO3 and degradation, whereas knockdown of Skp2 increases the quantity of FOXO3 protein. Acetylated lysine residues (K242, K259, K290, and K569) of FOXO3 have been mutated to arginine to mimic the deacetylated status of FOXO3 and finally lead to augmented Skp2 binding and inhibition of FOXO3 ubiquitination. By investigating the livers of mice deficient in SIRT1, increased expression of FOXO3 was detected, indicating SIRT1 regulates FOXO3 protein levels in vivo. Furthermore, elevation of SIRT1 and Skp2 expression in malignant PC3 and DU145 prostate cells can be explained by the downregulation of FOXO3 protein levels in these cells, which highlights that Skp2 ubiquitination and degradation of FOXO3 is acetylation-dependent [117].

### 3.8. DUB Enzymes Facilitate Skp2 Function

Recent studies have suggested that USP18 (ubiquitin specific peptidase 18) increases Skp2 mRNA expression by promoting Akt activation. Breast cancer patients with high expression of both USP18 and Skp2 had the worst survival rates, which suggested that USP18/Skp2 may act as a potential biomarker in breast cancer. [118]. Additionally, USP10 is one of the Skp2 deubiquitylation enzymes, which modulates the Skp2/Bcr-Abl axis by stabilizing Skp2 in chronic myeloid leukemia (CML) and thus promotes cell proliferation in CML cells. Targeting USP10/Skp2 may help overcome imatinib resistance in CML patients [16,61]. Studies also shows USP10, USP13, and USP14 bind with Skp2; however, the biological consequences of this binding await further exploration. 

### 3.9. Other Mechanisms

Drug resistance is mainly related with upregulated multi-drug resistance associated protein (MRP) and multi-drug resistance protein 1 (MDR1) expression. Interestingly, Skp2 siRNA inhibits the degradation of p27and down-regulates the expression of MRP in HL-60 (acute myeloid leukemia) adriamycin-resistant cells [72]. Over-expression of family with sequence similarity 60A (FAM60A) in A549 cisplatin-resistant cells led to Skp2 upregulation and enhanced cisplatin resistance in cancer cells, along with elevated MDR1 expression, and decreased caspase 3, cleaved-caspase 3, and caspase 8 expression, and further inhibited cancer cell death. Skp2 inhibition may reverse cisplatin resistance by eliminating the expression of FAM60A [47]. Some reports in the literature also reported that miR-21-5p, miR-26-5p, and miR-30-5p could downregulate Skp2 in both MCF-7 and tamoxifen-resistant MCF-7 cells and miR-339 decreases Skp2 expression in lung cancer cell line A549 [51,119].

## 4. Inhibitors Targeting Skp2 Overcome Resistance

We have already summarized some of mechanisms by which Skp2 contributes to chemoresistance; it seems that Skp2 plays its role through both its proteolytic function (E3 ligase activity) and non-proteolytic function (protein–protein interaction and epigenetic modifications). Destruction of Skp2 functions is emerging as a promising way to overcome drug resistance. Thus far, there have been many successful discoveries and developments of Skp2 inhibitors which were reviewed in [120], we also updated developments of Skp2 inhibitors in drug resistance as follows.

### 4.1. Targeting Skp2 Expression

With inhibition of the neddylation of Cullin1 and Ubc12, flavokawain A (FKA), flavokawain B (FKB), and gartanin induced Skp2 degradation, which inhibited cell growth and induced autophagy in prostate cancer [121,122,123]. All-trans retinoic acid (ATRA) induces ubiquitylation and degradation of the F-box protein Skp2 in Cullin1 independent manner. Stabilization of p27 results from the elimination of the F-box protein Skp2 by an ATRA-inducible ubiquitylation pathway [124]. SMIP004, was found to downregulate Skp2 and to stabilize p27, although it is not a proteasome inhibitor [125]. A novel selenonucleoside (4′-selenofuranosyl-2, 6-dichloropurine, LJ-2618) may bear results in overcoming paclitaxel resistance by promoting Skp2 degradation and stabilizing p27 expression in paclitaxel-resistant prostate cancer [45]. A γ-secretase inhibitor, DAPT, selectively depleted CD133^+^ cells, suppressed activated NOTCH1 and Skp2, induced p27, inhibited ACC growth in vivo, and sensitized CD133^+^ cells to radiation [65]. Gartanin docked onto the NEDD8-activating enzyme (NAE) complex, which results in inhibition of neddylation while inducing proteasomal-dependent Skp2 degradation [123].

### 4.2. Targeting Skp2-SKP1 Interaction and SCF Formation

Chan et al., identified 25 compounds that potentially interact with Skp2. Further experiments showed that compound #25 (C25), indeed, binds to Skp2, prevents Skp2-SKP1 interaction, and inhibits Skp2-SCF E3 ligase activity, which also inhibited Skp2-mediated p27 ubiquitination and Skp2-mediated Akt ubiquitination in vivo and in vitro. This consequently suppresses the survival of cancer cells and cancer stem cells [126]. C25 combined with bromocriptine (agonist of the dopamine D2 receptor) led to maximal apoptosis of human prolactinoma cells [127]. CpdA prevented incorporation of Skp2 into the Skp2-SCF ligase, and induced G1/S cell-cycle arrest, as well as Skp2-p27 dependent cell killing. By inhibition of p27 ubiquitination, and stabilization of p27 in vitro, CpdA increased the sensitivity of multiple osteosarcomas to dexamethasone, DOX, melphalan, and BTZ, and synergistically acted with this proteasome inhibitor [88]. Malek et al., identified a completely unique Skp2 inhibitor, DT204, which reduces Skp2 binding to Cullin-1 and Commd1 and that, in co-treatment with BTZ, overcomes drug resistance in multiple myeloma [52]. DT204 co-treatment with BTZ overcomes BTZ resistance and reduces the in vivo growth of myeloma tumors in murine models with survival benefits. Taken together, the results provide a proof of concept for rationally designed drug combinations that incorporate Skp2-SCF inhibitors to treat BTZ resistant disease [52,128].

### 4.3. Targeting the Interactions between Skp2 and Cks1

Cks1 bridges the interaction between Skp2 and p27 and facilitates p27 ubiquitination. Vorinostat enhances the protein stability of p27 and p21 by negative regulation of Skp2 and Cks1 in human breast cancer cells [68]. NSC689857 and NSC681152 disrupt the protein–protein interaction between Skp2 and Cks1 [129]. Linichlorin A and gentian violet also inhibits the interaction between Skp2–Cks1 and p27, thus inhibiting the ubiquitination and degradation of p27 in HeLa cells, which indicates a potential strategy for restoring p27 levels in human cancers [130]. 

### 4.4. Targeting the Binding Interface for p27

Wu et al., identified a set of small molecule inhibitors called as SKPins, which are specific to Skp2-SCF activity, using in silico screens targeted at Skp2-Cks1-p27 [131]. 1,25-(OH)_2_D_3_ mediates an increase in cyclin dependent kinase inhibitor p27 levels, inhibition of Cdk2 activity, hypophosphorylation of retinoblastoma protein, and accumulation of cells in G1. 1,25-(OH)_2_D_3_ reduces Skp2 expression level and decreases levels of Thr187 phosphorylated p27, which results from the translocation of CDK2 from the nucleus to cytoplasm, which is consistent with inhibition of Thr187 phosphorylation-dependent p27 degradation [132].

### 4.5. Targeting the Binding between Skp2 with p300

M1 blocks the interaction of Skp2 with p300, and thus facilitates p53 acetylation by p300 and promotes p53-mediated apoptosis [133]. 

In addition, it has also been reported that a variety of natural compounds, such as curcumin [23,54,134], quercetin [134], lycopene [134], epigallocatechin-3-gallate [135], vitamin D3 [132,136], dioscin [137], gartanin [123], and flavokawain A [35,122,138,139,140] inhibit the expression of Skp2 and subsequently exert anti-tumor activity.

However, these Skp2 inhibitors still needs further in vitro cell experiments and in vivo animal models to verify their anti-drug resistance effect.

## 5. Conclusion and Perspective

Skp2, as an E3 ligase, conjugates both K48-linked and K63-linked ubiquitin chains on its substrates, mediating proteasome degradation, as well as nonproteolytic degradation. The large protein–protein interaction network, centered by Skp2, underscores that Skp2 finely tunes oncogenesis. Normally, Skp2-linked K63-ubiquitination stabilizes and activates proteins, as demonstrated by Akt stabilization and NBS1 activation, leading to increased cell proliferation and enhanced DNA damage response, which contributes to drug resistance [79,100]. Nevertheless, Skp2 triggers K48-linked ubiquitination and degradation of cell cycle inhibitors, p27 and p21 [80]. In line with these experimental observations, the negative correlation between Skp2 and p27 was clinically viewed as an accurate prognostic marker of response to chemotherapy and overall survival in patients, which suggests that Skp2 may have important clinic benefits in this regard [44,85].

Post-translational modification had a great influence on Skp2 expression and activity, and further contributed to drug resistance. Phosphorylation status of the NLS domain of Skp2 determines protein activation and nucleus/cytoplasm location [76]. Acetylation of Skp2 by p300 increases Skp2 protein stability, and deacetylation by SIRT1/2/3 (sirtuin 1/2/3) promotes its ubiquitination and increases p27 protein half-life [101,104]. Recent studies have shown that multiple DUB enzymes bind with Skp2, among which USP18 and USP10 were shown to augment Skp2 function, but the consequences of other interactions about drug resistance remain elusive [16,61,118]. Of note, the cytoplasm translocation of Skp2 stabilizes Twist and degrade E-cadherin, leading to EMT, suggesting that Skp2 contributes to tumor migration and invasion [69,101,109,110].

The degradation of cell cycle proteins p27, cyclin E, and cyclin D is tightly controlled by Skp2 with sequential timing complexity. Accumulation of both p27 and cyclin E is observed in Skp2 deficient cells during G1/S transition, whereas CDK2-cyclin E associated kinase activity remains unchanged, despite p27 being an inhibitor of CDK2 activity. The binding between p27 and cyclin E is prerequisite for the degradation of p27. Although ubiquitination of p27 requires binding to CDK2-cyclin E, p27 inhibit phosphorylation of cyclin E and its subsequent ubiquitination. Predictably, unrestricted cyclin E expression and subsequent CDK2-cyclin E activation may be observed in cancers with p27 deletion or mutation. The dysregulation of cyclin E may bear pro-tumor properties [95]. Additionally, side effects resulting from enhanced cyclin D1 expression upon Skp2 inhibition warrant careful evaluation in the future as cyclin D1 is strongly implicated in mammary oncogenesis [96]. 

Over-expression of Skp2 highly correlated with drug resistance. Skp2 appears to be a promising target for overcoming drug resistance in cancer therapy. Thus far, multiple efforts have been made to develop novel inhibitors targeting the Skp2, which will benefit cancer patients in the future [120]. However, more clinically relevant human tumor models, such as PDX and organoids and genetic mouse models should be applied to carefully evaluate the efficacy of Skp2 inhibitors.

Nowadays, some new target-based degradation (TBD) strategies developed to inhibit over-expression of target proteins may overcome chemoresistance in cancer, these include proteolysis targeting chimeras (PROTAC) [141], affinity-directed protein missile system (AdPROMs) [142], biological PROTACs (bioPROTAC) [143], molecular glues [144,145], selective estrogen receptor down regulators (SERDs) [146], etc. These techniques can be applied to induce drug-resistance-cause protein degradation through the ubiquitin proteasome pathway by recruiting an E3 ligase to ligate the target protein for degradation. For instance, it has been reported that E3 ligase cereblon (CRBN) is highly expressed in lung cancer; thus, ligands for CRBN (lenidomide, thalidomide, pomalidomide) could be mounted in the PROTAC model to develop an anti-cancer therapy [147]. Likewise, the over-expression of Skp2 is observed in multiple cancers, as such the ligands for Skp2 in these cancers could be accommodated to target Skp2. Molecular glue compounds could induce protein–protein interactions and lead to protein degradation in the context of ubiquitin ligase. The known molecular glue degraders bind to the substrate receptor of E3 ubiquitin ligase and recruit target proteins for further degradation. It was demonstrated that the binding of thalidomide analogs (IMiDs) to CRL4^CRBN^ E3 ligase causes recruitment of selected zinc finger transcription factors, which leads to their ubiquitination and subsequent proteasomal degradation [148,149,150,151]. The molecular glue degraders of thalidomide analogs and aryl sulfonamides have been proved to be effective and are widely used in clinics. Therefore, the development of a new molecular glue degrader of Skp2 is very promising. Designing CRISPR/Cas9-based cancer epigenetic regulators in a more specific manner is a new trend that has caused great interest in recent years. However, CRISPR-based epigenome editors (CRISPR epi-editors), which consist of dCas9 and epigenetic effectors (fused or non-covalently) are being given serious considerations as a practical approach in cancer gene therapy, as they may activate tumor suppressor genes and inhibit tumor driving genes at the same time [152,153,154,155]. 

Conclusively, these new techniques have great advantages. Firstly, degradation can be induced via protein–protein interactions and epigenetic modifications, therefore, substrates are more specifically degraded. Secondly, the TBD model triggers degradation cascades and inhibits protein synthesis, thus blocking protein over-expressing during drug treatment. In the future, new strategies for targeting Skp2 in the treatment of cancer may be developed and we believe our review provides an updated view of Skp2 in chemoresistance and will inspire further studies in this field.

## Data Availability

Not Applicable.
